# Bortezomib and low dose venetoclax therapy for relapsed/refractory B-cell acute lymphoblastic leukemia with *IDH1* mutation

**DOI:** 10.1016/j.htct.2024.04.131

**Published:** 2024-09-20

**Authors:** Pronamee Borah, Ayoniza Maitri, Rahul Naithani

**Affiliations:** a Paras Health, Gurugram, India; b Jawaharlal Nehru Medical College and Hospiatal, Bhagalpur, India

*Dear Editor*,

The outcome of relapsed refractory acute lymphoblastic leukemia (ALL) is dismal. There is a need to identify novel therapies for this population.

A 63-year-old male was diagnosed with B-cell ALL. He received the Berlin-Frankfurt-Munster-95 (BFM-95) induction protocol and had induction failure. His blasts were dim/negative for CD19, CD20, and CD22. He received dexamethasone and hyperfractionated cyclophosphamide along with daratumumab and attained minimal residual disease (MRD)-negative remission after four weeks. He subsequently underwent a haploidentical hematopoietic stem cell transplant (HSCT). One year later, he relapsed with bright CD38^+^ blasts and even after three doses of daratumumab there was no response. A bone marrow aspirate revealed 35 % blasts that were negative for CD20; the CD19 and CD22 expressions were dim. Next-generation sequencing revealed an isocitrate dehydrogenase 1 (*IDH1*) mutation with a variable allele frequency of 28.5 %. At this stage, he was started on bortezomib at a dose of 1.3 mg/m^2^ IV once a week, dexamethasone 40 mg once a week along with venetoclax 100 mg from Day 1–7. Posaconazole prophylaxis was started but withheld on the days bortezomib was administered. The venetoclax dose was modified to 100 mg once a day due to a drug interaction. A bone marrow aspirate after four weeks revealed 10 % blasts in bone marrow with an MRD of 2.9 %. A second cycle was administered with the dose of venetoclax increased to 14 days of the four-week cycle with weekly bortezomib administration. A bone marrow evaluation after one month revealed an MRD of 0.01 %. The patient was unwilling to undergo a second HSCT and prophylactic cranial irradiation and he could not be enrolled in ongoing CAR-T cell therapy, therefore, bortezomib plus venetoclax and dexamethasone were maintained. After 12 weeks of therapy, his hypertension worsened to 190/105 mmHg with proximal myopathy and recurrent gastric upset and, as he also developed neuropathy and diarrhea, the steroids and bortezomib were stopped. Ten days later, when his platelet count started to drop, a bone marrow evaluation revealed 40 % blasts.

Bortezomib, as a single agent, has proven to be ineffective in adult ALL. However, in 5/7 children with T-cell ALL in combination with chemotherapy it yielded responses, albeit with some toxicity.^1^ Earlier, in a small series of three patients with relapsed/refractory early T-cell precursor ALL, 2/3 patients responded to a combination of bortezomib and venetoclax (Table 1).^2^ These patients received four doses of bortezomib with venetoclax being administered at a dose of 800 mg. Another case was reported where *IDH1*-mutated ALL was treated with venetoclax plus methotrexate and pegasparaginase.^3^ The emerging role of venetoclax-based treatments in ALL has recently been discussed even though the majority of patients enrolled have been of T-cell ALL.^4^ Canani et al. described responses in six patients with B-ALL using a venetoclax based regime; one patient in this cohort was treated with a bortezomib and venetoclax combination.^5^ At a later stage of therapy, tolerance was an issue with the current patient. Despite this, we are able to document a MRD negative remission in a B-ALL patient with the *IDH1* mutation. This short lived 12 week remission does provide a window to proceed with HSCT with curative intent.

**Table 1:** Recent publications on use of bortezomib in acute lymphoblastic leukemia.

Reference	Study population	Therapy	Response	Duration of response	Conclusion
1	30 children with B-cell precursor ALL 7 children with T-cell ALL Median age 10.7 years	Bortezomib (1·3 mg/m^2^/dose) on Days 1, 4, 8 and 11 And conventional chemotherapy	23 CR 4 partial response	2-year disease free survival: 27.7 %	Bortezomib with chemotherapy is a suitable/effective option for treating refractory ALL.
2.	3 Adults ETP-ALL	Venetoclax (800 mg/day) for 28 days Bortezomib 1.3 mg/m^2^ twice a week (in 2 patients) or 4 weeks (in 1 patient)	1 CR 2 partial remission	8 months	Effective and well-tolerated chemotherapy-free strategy for R/R T-ALL
3.	60 Year-old male ALL with *IDH1* mutation	Venetoclax (400 mg/day) for 28 days And MPV chemotherapy	CR	Died 1 year after HSCT due to pulmonary aspergillosis	Needs further validation
4.	Systemic review	Venetoclax with chemotherapy	variable	variable	Venetoclax containing regimens are relatively safe and can achieve deep responses, although these are frequently transient.
5.	B-cell ALL 6 T-Cell ALL 11 Median age 32 years	Venetoclax + chemotherapy and/or navitoclax	CR in 5 patients CRi in 3 patients Partial response 3 patients (2 CRi and 3 MLFs) No response in 7 patients	Progression free survival: 5 Months	Ven-chemotherapy (55 %) and vennavitoclax (46 %) in RR ALL are efficacious with moderate toxicity

T-ALL: T-cell acute lymphoblastic leukemia; ALL: Acute lymphoblastic leukemia; ETP: Early T cell precursor; RR: Relapse/refractory; HSCT: hematopoietic stem cell transplant; CR: complete response; CRi: complete response with incomplete blood recovery; MLF: Morphologic Leukemia-free state.

## Authors' contributions

All authors contributed to the study's conception and design. Material preparation, data collection and analysis were performed by PB, AM and RN. All authors read and approved the final manuscript.

## Compliance with ethical standards

Pronamee Borah declares that she has no conflict of interest. Rahul Naithani declares that he has no conflict of interest. Ayoniza Maitri declares that she has no conflict of interest.

## Ethical approval

This article does not contain any studies with human participants or animals performed by any of the authors.

## Conflicts of interest

None.

## Data Availability

The data used for this research are available from the corresponding author upon reasonable request.
